# Levels and Factors Associated with Resilience in Italian Healthcare Professionals during the COVID-19 Pandemic: A Web-Based Survey

**DOI:** 10.3390/bs10120183

**Published:** 2020-11-29

**Authors:** Lucia Lisi, Jacopo Ciaffi, Antonella Bruni, Luana Mancarella, Veronica Brusi, Pasquale Gramegna, Claudio Ripamonti, Elisabetta Quaranta, Elena Borlandelli, Gaetano Gallo, Eugenio Garofalo, Agostino Chiaravalloti, Pasquale Viola, Piero Ruscitti, Giacomo Caio, Martina D’Onghia, Andrea D’Amuri, Antonio Cimellaro, Giancarlo Facchini, Micaela La Regina, Luca Spinardi, Roberto De Giorgio, Roberto Giacomelli, Maria Paola Landini, Domenico Berardi, Riccardo Meliconi, Francesco Ursini

**Affiliations:** 1 Medicine & Rheumatology Unit, IRCCS Istituto Ortopedico Rizzoli, 40136 Bologna, Italy; lucia.lisi@ior.it (L.L.); luana.mancarella@ior.it (L.M.); veronica.brusi@ior.it (V.B.); pasquale.gramegna@ior.it (P.G.); claudio.ripamonti@ior.it (C.R.); elisabetta.quaranta@ior.it (E.Q.); riccardo.meliconi@ior.it (R.M.); francesco.ursini2@unibo.it (F.U.); 2 Neuropsychiatric Rehabilitation, Villa Patrizia Hospital, 10045 Piossasco, Torino, Italy; bruni_antonella@yahoo.it; 3 Radiology Unit, Department of Experimental, Diagnostic and Specialty Medicine, S. Orsola Malpighi University Hospital, 40138 Bologna, Italy; elenaborlandelli@libero.it; 4 Department of Medical and Surgical Sciences, University of Catanzaro, 88100 Catanzaro, Italy; gaetanogallo1988@gmail.com; 5 Anaesthesia and Intensive Care Unit, Department of Medical and Surgical Sciences, University of Catanzaro, 88100 Catanzaro, Italy; eugenio.garofalo@gmail.com; 6 Department of Biomedicine and Prevention, University Tor Vergata, 00133 Rome, Italy; agostino.chiaravalloti@gmail.com; 7 IRCCS Neuromed, 86077 Pozzilli, Italy; 8 Audiology Unit, Department of Experimental and Clinical Medicine, University of Catanzaro, 88100 Catanzaro, Italy; pasqualeviola@unicz.it; 9 Division of Rheumatology, Department of Biotechnological and Applied Clinical Sciences, University of L’Aquila, 67100 L’Aquila, Italy; piero.ruscitti@univaq.it (P.R.); roberto.giacomelli@cc.univaq.it (R.G.); 10 Internal Medicine Unit, Department of Morphology, Surgery and Experimental Medicine, University of Ferrara, 44121 Ferrara, Italy; caigmp@unife.it (G.C.); martina.donghia@gmail.com (M.D.); dgrrrt@unife.it (R.D.G.); 11 Internal Medicine Academic Unit, Department of Medical Sciences, University of Ferrara, 44121 Ferrara, Italy; dmrndr@unife.it; 12 Internal Medicine Unit, Pugliese-Ciaccio Hospital, 88100 Catanzaro, Italy; antocime@hotmail.it; 13 Diagnostic and Interventional Radiology Unit, IRCCS Istituto Ortopedico Rizzoli, 40136 Bologna, Italy; giancarlo.facchini@ior.it; 14 Internal Medicine & Clinical Risk Management Unit, ASL5, 19121 La Spezia, Italy; micaela.laregina@asl5.liguria.it; 15 Diagnostic and Interventional Neuroradiology Unit, S. Orsola Malpighi University Hospital, 40138 Bologna, Italy; luca.spinardi@aosp.bo.it; 16 Scientific Direction, IRCCS Istituto Ortopedico Rizzoli, 40136 Bologna, Italy; mariapaola.landini@ior.it; 17 Department of Biomedical and Neuromotor Sciences (DIBINEM), University of Bologna, 40123 Bologna, Italy; domenico.berardi@unibo.it

**Keywords:** COVID-19, resilience, depression, anxiety, healthcare professionals

## Abstract

Background: Resilience is defined as the capacity to cope successfully with change or adversity. The aims of our study were to investigate levels of resilience in Italian healthcare professionals (HCPs) during the Coronavirus disease 2019 (COVID-19) pandemic and to identify potential predictors of resilience. Methods: We performed a web-based survey of HCPs (*n =* 1009) working in Italian hospitals during the COVID-19 pandemic. The survey contained a 14-item resilience scale (RS14) and questionnaires to evaluate depression and anxiety symptoms. Non-HCP individuals (*n =* 375) from the general population were used for comparison. Results: HCPs showed significantly lower resilience compared to the control group (*p* = 0.001). No significant differences were observed after stratification for geographical area, work setting, role, or suspected/confirmed diagnosis of COVID-19. In a linear regression analysis, RS14 was inversely correlated with depression (R^2^ = 0.227, *p* < 0.001) and anxiety (R^2^ = 0.117, *p* < 0.001) and directly correlated with age (R^2^ = 0.012, *p* < 0.001) but not with body mass index (BMI, R^2^ = 0.002, *p* = 0.213). In male HCPs, higher depression score (odds ratio (OR) 1.147, *p* < 0.001) or BMI (OR 1.136, *p* = 0.011) significantly predicted having low resilience. In female HCPs, higher depression score (OR 1.111, *p* < 0.0001) and working in a COVID-19 free setting (OR 2.308, *p* = 0.002) significantly predicted having low resilience. HCPs satisfied with personal protective equipment had higher levels of resilience (*p* < 0.010). Conclusions: Our findings suggest that resilience was lower in Italian HCPs than in the general population after the first COVID-19 wave. Specific factors can be identified, and targeted interventions may have an important role to foster resilience of HCPs.

## 1. Introduction

Several interpretations of the term “resilience” have been reported in the literature [1]; however, a unifying synthetic definition is that resilience is “the psychological phenomenon representing the capacity of individuals to cope successfully with significant change, adversity or risk” [2,3]. Consequently, the main premise of resilience is deemed to be adversity, and the main consequence is the positive adaptation to it [3]. Furthermore, resilience is the capacity to not only overcome life’s challenges but to learn and grow from them and become stronger as a result of such challenges [3]. This ability is then a key factor in determining how people will respond to unanticipated change, and it can develop over time under the right circumstances [3,4]. In particular, the literature suggests that resilient individuals are better equipped to deal with the stressors that result from a constantly demanding work environment, such as health services [3].

In recent months, humanity has faced the emergence of an unexpected infectious threat. Since its first appearance in Wuhan, China, in December 2019 [5], the novel severe acute respiratory syndrome coronavirus 2 (SARS-CoV-2) had rapidly spread over all continents, and the World Health Organization (WHO) officially declared the pandemic emergency on 11 March 2020 [6]. Immediately, the disease—namely Coronavirus Disease 19 or COVID-19—exhibited devasting power on society, with more than 56 million people infected and over 1.3 million deaths as of 18 November 2020 [7].

Extraordinary social containment measures with total lockdown of most involved countries had to be introduced, with a consequent deep, worldwide, economic crisis [8]. Intuitively, these circumstances affected the psychological wellness of people, promoting a sudden increase in mental health problems such as depression, anxiety, and post-traumatic stress disorder (PTSD) [9,10,11].

Healthcare professionals (HCPs) represented, from the earliest phases of the pandemic, one of the most vulnerable categories. National physician associations periodically released war-dead bulletins; in Italy—the first country to be hit hard in Europe—200 medical doctors and 41 nurses died as a direct consequence of dealing with the infectious plague in a context of inadequate health system organization and shortage of protective equipment [12]. In addition to this stunning death toll, the COVID-19 pandemic put HCPs under an unprecedented physical and emotional strain. Working under extreme pressures, prolonged shifts due to insufficient staffing and incoming sickness of colleagues, insufficient availability of personal protective equipment (PPE), fear of infection and transmission to family members in lack of evidence-based life-saving treatments, and the daily need to take morally burdensome decisions (e.g., how to distribute insufficient resources/treatments between equally needy patients), resulted in a profound sense of danger, moral injury and mental health problems [13,14,15,16]. The burden of such a stressful work environment could represent an additional threat to the physical and mental wellbeing of HCPs, leading to a higher risk of burnout and traumatic-stress-like symptoms. In normal circumstances, HCPs can efficiently counteract these adversities by positive individual and relational coping strategies (e.g., problem-focused coping, taking time out, and giving and receiving support from co-workers) [17]. However, the immediacy of the COVID-19 menace makes it possible to hypothesize a profound interference with the possibility to put these coping strategies into practice.

In this regard, a recent meta-analysis of studies investigating the main psychological consequences of the COVID-19 pandemic—including 19 studies conducted among HCPs—demonstrated a pooled prevalence of 26% for anxiety and 25% for depression [18]. It is well known that high levels of depression and anxiety weaken resilience and influence coping style [19], making it more or less maladaptive. Furthermore, sleep disturbances have been reported in 39% of HCPs during the COVID-19 pandemic [20]. It is expected that these negative stress-related outcomes can impact not only the wellbeing of the HCPs, but also their ability to care effectively for others [21].

Social (e.g., social activities, family support), personal (e.g., leisure time) and lifestyle factors (e.g., physical activity), alongside work environment (e.g., experience, control over time, content of work, supportive colleagues) and personality features (e.g., optimistic, persevering, and cooperative), have been shown to contribute to the development of resilience in HCPs [22,23,24]. Moreover, sociodemographic factors such as lower body mass index (BMI) [25,26,27] can also have a positive impact on resilience and mood or anxiety disorders.

In this background, we hypothesized that, as a consequence of COVID-19 pandemic, some of these mechanisms could be hampered in HCPs, breaking down the protective mechanism of raising resilience, and that this phenomenon could be even more pronounced when factors such as increased exposure to risk, lack of adequate PPE or excessive working hours are present.

To address this issue, we investigated levels of resilience in Italian HCPs during the COVID-19 pandemic in comparison with individuals not involved in healthcare to ascertain the harmful/protective effect of surviving this event on the front lines. Secondly, hypothesizing that specific factors could have an impact on resilience, we analyzed levels of resilience according to the HCP’s role, work setting, or geographical area. Finally, we assessed potential determinants of low resilience in our study sample in order to hypothesize targets for resilience-enhancing interventions to be adopted in case of future unexpected pandemic events. Moreover, as gender differences were outlined in levels of resilience and coping strategies between men and women [28,29], we stratified our study in order to identify gender-specific resilience-affecting factors.

## 2. Materials and Methods

### 2.1. Social and Health Context and Design of the Study

To investigate the levels and determinants of resilience in healthcare settings, we performed a cross-sectional survey on Italian HCPs. During the first wave of the SARS-CoV-2 pandemic, Italy was one of the hardest-hit countries, with early COVID-19 cases appearing immediately after the primary outbreak in China and exponential growth of daily deaths peaking around 1000 at the end of March 2020 [30]. This unexpected burden put an unprecedented strain on the National Health System, forcing HCPs to work under extremely stressful conditions of insufficient availability of PPE and prolonged shifts due to insufficient staffing or incoming sickness of physicians and nurses. The unpreparedness of the health system resulted in >20,000 cases of infection in HCPs at the end of April 2020 [31].

Data were collected between 22 and 31 May 2020, shortly after the peak of the pandemic in Italy, through a web-based survey using the Google Forms platform, available at https://docs.google.com/forms/. Google Forms is a free, web-based personalized survey administration tool that has been extensively used in medical research [32,33,34]. Reporting was compliant with the Checklist for Reporting Results of Internet E-survey (CHERRIES) [35].

### 2.2. Survey Development

A group of senior researchers (F.U., L.L., R.M., P.R., M.P.L., R.G.), including a medical psychotherapist (L.L.), designed the survey draft. The content was further reviewed by all study researchers, including two psychiatrists (A.B., D.B.). Furthermore, pilot testing investigating the understandability of questions was performed on a pool of physicians (*n* = 30) and nurses (*n* = 20) who did not participate in developing the survey. The final version was consequently modified following their suggestions and approved by consensus.

The survey was found to require a total of 15 min to be completed. Results were transmitted to the database only if the participant clicked on “survey completed” at the end of the questionnaire. Questions were listed in the same order for all participants. Participants were able to return to previous sections to modify their answers before submission. The survey could not be submitted unless completed in all its parts since all questions were mandatory.

### 2.3. Survey Structure

The survey consisted of four different sections including a total of 68 questions. Questions were preceded by a preface stating the overall goal of the survey, the names and affiliations of research group members, and that collected data were explicitly used for research purpose and publication. Details of the survey structure are reported in Table S1.

In Section 1, questions regarded demographic characteristics, work setting, specific healthcare profession (including, if medical doctors, specialty area), details on testing for COVID-19 and disease development, satisfaction with protective equipment provided by workplace, smoking status, concurrent diseases and medications used. The section was composed of 19 individual questions, of which the majority were presented as multiple choice (9 questions) with only one selection allowed, followed by free-text answers (6 questions), check-boxes (2 questions) with more than one selection allowed, list (1 question) or, only for PPE satisfaction, a 5-point Likert-type scale (1 question).

In Section 2, participants were asked to reply to the 14-item Resilience Scale (RS14) [36]. RS14 is a self-administered scale evaluating the main characteristics of the resilience core. RS14 has been validated in several languages, including Italian [37]. Each item is scored on a 7-point Likert-type scale from 1 (strongly disagree) to 7 (strongly agree) with a total sum ranging from 14 to 98. Scores greater than 90 indicate high resilience, 82–90 moderately high, 65–81 moderately low to moderate, 57 to 64 low, and scores lower than 57 indicate very low resilience [36]. The Italian version of RS14 has been provided by Resilience Center, Worden (MT), USA, upon license.

In Section 3, participants were asked to reply to the 21 items of the Beck Depression Inventory-II (BDI-II) scale in its validated Italian translation [38]. The BDI-II is a widely used self-administered scale assessing the severity of depression in normal individuals or psychiatric populations [39]. Each item is scored on a 4-point scale ranging from 0 (symptom absent) to 3 (severe symptom) with a total sum ranging from 0 to 63. In non-clinical populations, a score > 20 has been considered suggestive of depression [40]. All questions of the scale were presented as multiple choice.

Finally, in Section 4, participants were asked to reply to the 14 items of the Hospital Anxiety and Depression Scale (HADS) in its validated Italian translation [41]. The HADS was originally developed to measure anxiety and depression in a general population of medical patients [42]. The questionnaire comprises seven questions for anxiety and seven questions for depression, scored independently (HADS-A and HADS-D score, respectively). Each item is scored 0 to 3, for a total score ranging from 0 to 21 for each domain. In the non-clinical population, a cut-off of >10 has been suggested to distinguish between cases and non-cases [43]. All questions were presented as multiple choice.

### 2.4. Target Population and Survey Administration

The target population comprised physicians and other HCPs (nurses, radiology technicians, laboratory technicians, podiatrists, physiotherapists, hospital biologists, psychologists) actively working in Italian public or private hospitals during the COVID-19 pandemic (January–May 2020). A total of ~5000 individual HCPs were directly contacted by the members of the research group and asked to participate in the survey on a voluntary basis. No monetary or non-monetary incentives were provided for the completion of the survey. Participants were invited via social media (Facebook), mobile phone messages, and email. Each participant was sent a unique link to provide access to the online survey page. One follow-up reminder message was sent during the study period; however, participants were explicitly asked to answer the survey only once. The only exclusion criterion was age < 18 years. A group of individuals living in Italy and not working in healthcare-related environments was recruited as a frame of reference. A total of ~2000 individuals were directly contacted by the members of the research group using the methodology reported above. The only exclusion criterion for this group was also age < 18 years.

### 2.5. Ethical Considerations

By voluntarily taking part in the survey, each participant explicitly authorized the use of the data recorded in the questionnaire for research purposes and their publication, as clearly stated in the questionnaire preface. The research was conducted in compliance with the Declaration of Helsinki and its latest amendments [44]. No personally identifiable information was collected, and data remained completely anonymous throughout the study. The study was approved by the local Ethics Committee (Comitato Etico Area Vasta Emilia Centrale, Bologna, Italy—Approval number: 0007795/2020).

### 2.6. Statistical Analysis

Data are expressed as mean ± standard deviation (SD), median (25th–75th percentile), or number (percentage) as appropriate. The internal consistency reliability of the RS14, BDI-II, HADS-A, and HADS-D in our sample was evaluated using Cronbach’s alpha coefficient [45]. A value ≥ 0.70 is usually considered acceptable. The Student’s T test was used for comparing means of continuous variables between two groups. The Analysis of Variance (ANOVA) with Bonferroni’s correction for multiple comparisons was used to reject the null hypothesis of equality of means when comparing continuous variables between multiple groups. Statistical assumptions, including normality of data and homogeneity of regression slopes, were assessed, and if assumptions were not met, appropriate transformations were employed. The Shapiro–Wilk test was used to assess normality [46]. Highly skewed variables were thus ln-transformed before analysis. Fisher’s exact test was used to compare categorical variables. Univariate linear regression models were built to evaluate the correlation between continuous variables, expressed as R^2^ coefficient and relative *p*-value. Univariate and multivariate logistic regression models were built to assess the predictivity of continuous or categorical variables for the dichotomic dependent variable “low resilience”, expressed as odds ratio (OR) and 95% confidence interval (95% CI). All tests were two-tailed. All analyses were performed using SPSS^®^ software Version 26 (IBM Corp. Released 2019. IBM SPSS Statistics for Windows, Version 26.0. Armonk, NY, USA). Graphs were created using PRISM software, Version 8 (GraphPad Prism version 8.4.2 (679) for Windows, GraphPad Software, La Jolla, CA, USA).

## 3. Results

### 3.1. General Characteristics of the Study Sample

General characteristics of the study sample are reported in Table 1.

On a total of >5000 individual HCPs contacted by members of the research group, 1009 replied to the survey. Of these, 195 (19.3%) worked in COVID-19-free settings, 451 (44.7%) were indirectly involved in COVID-19 care (e.g., working in COVID-dedicated hospital but not in COVID-dedicated wards), while 363 (36.0%) were working in COVID-dedicated wards. Regarding controls, 375 out of >2000 individuals contacted replied to the survey. As mentioned in the methods section, no partial responses were allowed; therefore, all questionnaires were fully completed by respondents. In our sample, Cronbach’s alpha was 0.90 for RS14, 0.89 for BDI-II, 0.83 for HADS-A, and 0.79 for HADS-D.

In ANOVA analysis, significant differences were observed when comparing HCPs vs. controls regarding age (*p* < 0.0001), marital status (*p* < 0.0001), smoking habits (*p* = 0.001), geographical area (*p* < 0.0001), and presence of chronic comorbidities (*p* = 0.004). None of the control individuals underwent COVID-19 screening (*p* < 0.0001) or developed symptomatic COVID-19 (*p* < 0.0001).

When evaluating the HCPs group itself, more physicians and fewer “other HCPs” (e.g., laboratory technicians, physiotherapists, or psychologists) worked in direct or indirect COVID-19 care (*p* < 0.001); no significant differences were observed regarding the number of nurses. Physicians working in direct or indirect COVID-19 care were mainly clinical, surgical, intensive care specialists, and radiologists (*p* < 0.0001). Only 61.9% of HCPs working in direct or indirect COVID-19 care regularly underwent COVID-19 testing, and 12.4% of them developed symptomatic COVID-19 (*p* < 0.0001). Satisfaction for PPE supplied by employers was higher in physicians working in COVID-19 environments (*p* < 0.0001).

### 3.2. Levels of Resilience in HCPs during the COVID-19 Pandemic

**Figure 1:**
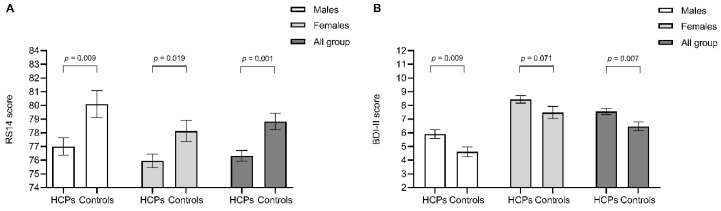

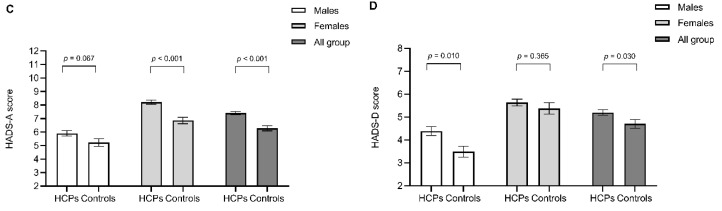
Resilience (**A**), depression (**B**,**D**), and anxiety (**C**) in healthcare professional (HCP) compared to control group. Data are expressed as mean and standard error of the mean and are stratified according to gender. RS14: 14-item Resilience Scale; BDI-II: Beck Depression Inventory-II scale; HADS-A: Hospital Anxiety and Depression Scale-Anxiety; HADS-D: Hospital Anxiety and Depression Scale-Depression.

HCPs showed significantly lower resilience, as measured by RS14, when compared to the control group (76.3 ± 12.2 vs. 78.8 ± 11.7, *p* = 0.001; Figure 1A). Details of responses to individual items of the RS14 questionnaire in HCPs and controls are reported in Table S2. Furthermore, HCPs showed higher levels of depression (BDI-II: 7.55 ± 6.91 vs. 6.47 ± 6.27, *p* = 0.007; HADS-D: 5.20 ± 3.82 vs. 4.70 ± 3.61, *p* = 0.030; Figure 1B,D, respectively) and anxiety (HADS-A: 7.40 ± 4.02 vs. 6.28 ± 3.60, *p* < 0.001; Figure 1C) when compared to controls. The prevalence of depression in HCPs was 5.8% and 9.6% with a cut-off of BDI-II > 20 or HADS-D > 10, respectively. The prevalence of anxiety was 22.6% with a cut-off of HADS-A > 10.

This difference in RS14 score between HCPs and controls was maintained after stratification for gender (*p* = 0.009 for males and 0.019 for females, respectively; Figure 1A). The prevalence of low resilience, defined as having an RS14 score ≤ 64, was significantly higher in HCPs vs. controls (15.3% vs. 11.5%, respectively; *p* = 0.042). Within the group of HCPs, no significant differences were found in the prevalence of low resilience between males and females (14.2% vs. 15.9%, *p* = 0.520).

BDI-II score and HADS-D were significantly higher in male HCPs vs. controls (*p* = 0.009 and 0.010, respectively; Figure 1B,D) but not in female HCPs, while HADS-A was higher only in female HCPs (*p* < 0.001; Figure 1C) but not in male HCPs. Within the group of HCPs, females showed levels of depression and anxiety higher than males (*p* < 0.001 for BDI-II, HADS-D, and HADS-A).

**Figure 2:**
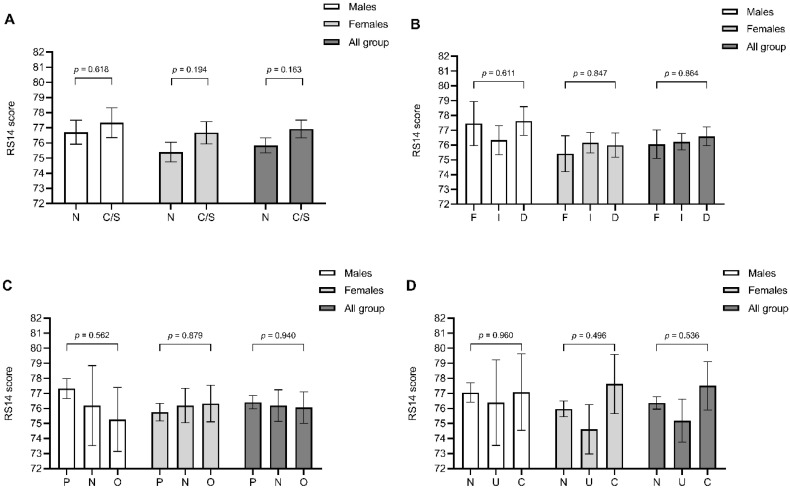
Levels of resilience measured through the 14-item Resilience Scale (RS14) in healthcare professionals after stratification for geographical area (**A**), work setting (**B**), role (**C**), and development of symptomatic COVID-19 (**D**). Data are expressed as mean and standard error of the mean and are stratified according to gender. In panel (**A**): N = Northern Italy, C/S = Central and Southern Italy. In panel (**B**): F = COVID-19-free setting, I = Indirect COVID-19 care, D = Direct COVID-19 care. In panel (**C**): P = Physicians, N = Nurses, O = Other roles. In panel (**D**): N = Never diagnosed with COVID-19, U = Uncertain symptoms of COVID-19, C = Confirmed diagnosis of symptomatic COVID-19.

No significant differences were observed in levels of resilience after stratification for geographical area (*p* = 0.163; Figure 2A), work setting (*p* = 0.864, Figure 2B), working role (*p* = 0.940; Figure 2C), or suspected/confirmed diagnosis of COVID-19 (*p* = 0.536; Figure 2D).

### 3.3. Factors Affecting Resilience in HCPs during the COVID-19 Pandemic

**Figure 3:**
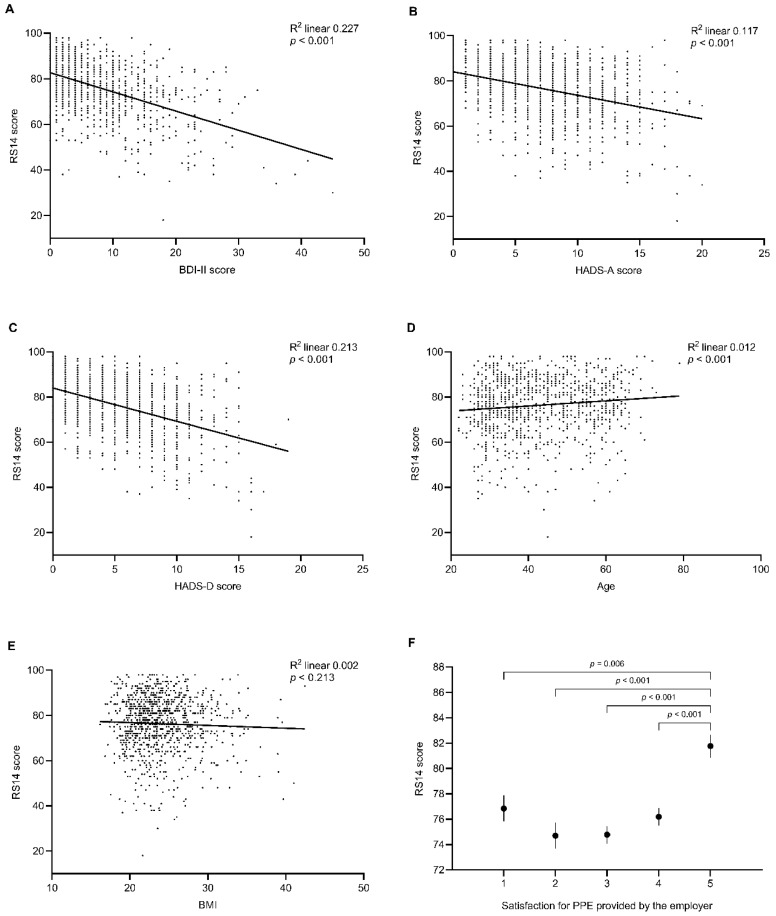
Linear regression (dot plot with overlayed regression line) showing the association between resilience and depression (**A**,**C**), anxiety (**B**), age (**D**), and BMI (**E**) in healthcare professionals. (**F**) shows levels of resilience in healthcare professionals after stratification for satisfaction for personal protective equipment. Data are expressed as mean and standard error of the mean. RS14: 14-item Resilience Scale; BDI-II: Beck Depression Inventory-II scale; HADS-A: Hospital Anxiety and Depression Scale-Anxiety; HADS-D: Hospital Anxiety and Depression Scale-Depression.

In univariate linear regression analysis performed in the HCPs sample, RS14 was inversely correlated with BDI-II score (R^2^ = 0.227, *p* < 0.001; Figure 3A), HADS-A score (R^2^ = 0.117, *p* < 0.001; Figure 3B), and HADS-D score (R^2^ = 0.213, *p* < 0.001; Figure 3C) and directly correlated with age (R^2^ = 0.012, *p* < 0.001; Figure 3D) but not with BMI (R^2^ = 0.002, *p* = 0.213; Figure 3E).

The association between resilience and PPE satisfaction, evaluated on a 5-point Likert-type scale, was investigated by comparing RS14 levels after stratification for PPE scoring. Only HCPs scoring 5 in PPE satisfaction showed higher levels of resilience compared to other groups (Figure 3F).

**Figure 4:**
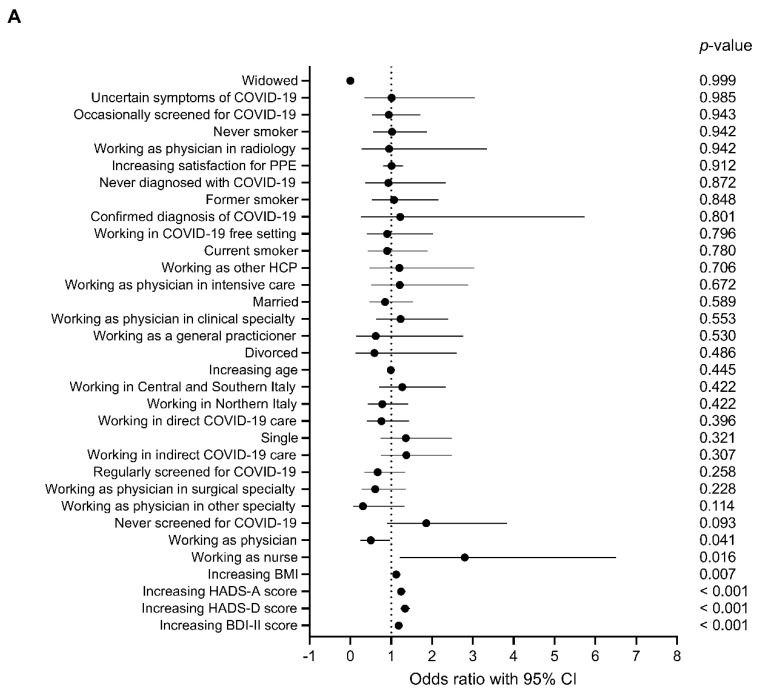

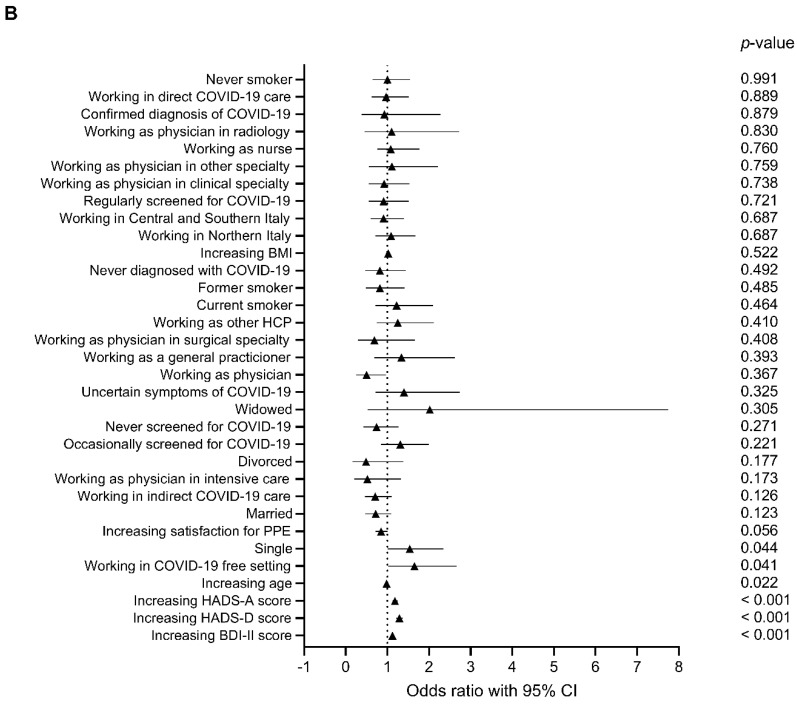
Logistic regression analysis of variables predicting low resilience in male (**A**) and female (**B**) healthcare professionals. Data are expressed as odds ratio and 95% confidence interval. Variables are sorted according to *p*-values.

Furthermore, we aimed to investigate the predictivity of selected variables on the odds of having low resilience. For this purpose, we performed univariate logistic regression analyses using RS14 score ≤ 64 (defining “low” to “very low” resilience according to the proposed cut-off [36]) as the dependent variable. In male HCPs, higher BDI-II, HADS-D, HADS-A score, or body mass index (BMI) and working as a nurse significantly increased the odds of having low resilience, while working as a physician was associated with lower probability of having low resilience (Figure 4A). In female HCPs, higher BDI-II, HADS-D, and HADS-A score, increasing age, working in COVID-19-free settings, and “single” marital status significantly increased the odds of having low resilience (Figure 4B).

Subsequently, to evaluate the independency of the predicting variables, we performed multivariate logistic regression analysis including the five strongest predictors of low resilience in both groups (classified on univariate logistic regression *p*-value), in order to maintain a variables-to-cases ratio of 1:10 and avoid model overfitting. To minimize redundancy of information, we decided to not include HADS-D in the analysis because it was significantly correlated with both BDI-II score (R = 0.77, *p* < 0.0001) and HADS-A score (R = 0.71, *p* < 0.0001).

In male HCPs, the logistic regression model including BDI-II, HADS-A, BMI, working as a nurse, and working as a physician showed that only higher BDI-II score (OR 1.147, 95% CI 1.074–1.225, *p* < 0.001) or BMI (OR 1.136, 95% CI 1.030–1.253, *p* = 0.011) significantly predicted having low resilience.

In female HCPs, the logistic regression model including BDI-II, HADS-A, age, single marital status, and working in a COVID-19-free setting showed that only higher BDI-II score (OR 1.111, 95% CI 1.070–1.154, *p* < 0.0001) and working in a COVID-19-free setting (OR 2.308, 95% CI 1.354–3.936, *p* = 0.002) significantly predicted having low resilience.

## 4. Discussion

Resilience is a multidimensional construct that explains how people who emphasize the adaptation to experiences perceived as threatening or traumatic may show positive effects in the long term [47].

Resilience positively affects physical health and improves survival and mental wellness, significantly reducing the risk of developing depression, anxiety, and stress. Obviously, the reverse paradigm also applies. High levels of depression and anxiety weaken resilience and influence coping style, making it more or less maladaptive [48]. Furthermore, recent studies have shown that resilience can ameliorate the perception of pain and emotional distress by promoting effective coping strategies with an overall positive effect in chronic and disabling diseases. Exposure to a disease, but also to the care of a person with severe, progressive, and incurable diseases, entails an emotional price and has an impact on quality of life [49].

In our study, we conducted a web-based survey to investigate resilience, and factors associated with it, in HCPs during the COVID-19 pandemic. In apparent contrast with the abovementioned basic principles, we found that HCPs had lower levels of resilience compared to a control group of individuals from the general population. Notably, levels of resilience were not affected by geographical factors (and therefore by their epidemiological correlates, e.g., different infection rates or clustering of cases), work setting (direct care of COVID-19 patients vs. working in a COVID-19-free setting), specific HCP role (physician vs. nurses vs. other HCPs), or having developed the disease itself. Overall, we feel that the difference between HCPs and controls may be mainly attributed to the significant increase in work burden, worsened by the absence of sufficient social and family support as a consequence of strict social distancing measures (lockdown), and to the concern of spreading the virus to family, friends or colleagues. From an individual perspective, the full awareness of the severity of the disease and a deeper understanding of the true risks (e.g., intensive care unit admission with invasive ventilation, long-term respiratory failure, or death) may result in more pervasive psychological consequences in HCPs compared to the community.

Indeed, Karasek’s theories (demands-control model of response to professional stress) highlight how the experience of stress (objective increase in workload, absence of adequate rest) and the individual perception of it (subjective discomfort related to the fear of contracting the disease and its possible consequences), in the absence of adequate social and family support (social distancing), influence the global reaction and the overall response strategies to the stressful event [50].

It is easily conceivable that HCPs may represent a group with a higher psychological vulnerability during the COVID-19 pandemic, as already suggested by recent articles [48,51]. In support of this theory, it must be pointed out that the theoretical model of stress emphasizes the cognitive processing of the stressful event and suggests that the thought and meaning of the event experienced by the individual are the main intermediaries of the long-term response to stress. During their task of treating patients when adequate resources are lacking, many HCPs experienced negative feelings of guilt and helplessness over being unable to help others. The attribution of responsibility, to oneself and to others, has a significant weight in the self-esteem of each element of the health team in routine contexts and is even amplified in this case of planetary emergency [49].

When analyzing factors associated with low resilience, we found a strong, independent, inverse relationship between resilience and symptoms of depression. This correlation is not surprising as it replicates a well-established mutual relationship as well as everyday experience in clinical practice [52].

In male HCPs, increasing BMI was the second independent predictor of low resilience, and this is consistent with previous literature suggesting an inverse correlation between BMI and resilience [25]. On the other hand, when observing the female sample, working in a COVID-19-free setting was identified as an independent predictor of having low resilience. It must be taken into account that, especially in the first weeks after the outbreak, working in a COVID-19-dedicated department was often a voluntary decision, making the working context a conscious and already-internalized a priori choice. This could have selected the more resilient HCPs and encouraged resilient coping styles. Such a result is not surprising in light of the abovementioned psychological principles on resilience. Sharing the problem and adopting behavioral and interventional protocols inspired by non-flexible safety standards help to generate awareness, knowledge of the problem, and therefore, control of negative emotions. Conversely, in contexts where people tend to act on the basis of individualistic principles and with poorly detailed rules, it is possible to hypothesize an increase in anxiety-provoking triggers. On the other hand, being “useful” to others may increase self-esteem and self-satisfaction and therefore encourage resilient coping styles.

Additionally, we feel that another result from our data deserves discussion: the relationship between satisfaction with PPE and resilience. First of all, the level of satisfaction with PPE was lower in HCPs working in COVID-19-free setting, thus suggesting the role of adequate protocols and organization in generating positive emotional feedback on HCPs. Secondly, the strong media resonance on the need for wearing PPE on the one hand improved adherence by the community but on the other hand made HCPs more critical regarding the quality, adequacy, frequency of distribution, and method of use of the same PPE in the workplace. Accordingly, in our data, only HCPs who considered themselves fully satisfied with the quality of the PPE provided by the employer showed significantly higher levels of resilience. This relationship was not linear and, for instance, did not occur in those who expressed an intermediate rating with respect to the perception of quality of PPE. HCPs are familiar with the protective properties of different PPE and acknowledge with good confidence that wearing an inadequate PPE does not provide a barrier from the risk of infection.

Our study is not without limitations. First, the design is cross-sectional, and data about levels of resilience in the same Italian HCPs cohort before or after the first viral outbreak were not available. As a consequence, we were unable to analyze the potential relationship between the emergent stressor and the change in psychological features or the process of development of coping strategies; accordingly, we cannot draw firm conclusions regarding the longitudinal change in resilience levels in response to the COVID-19 pandemic. Given the fact that resilience is a psychological resource that does not arise immediately but grows over time, it is possible that the traumatic experience was still too vivid in HCPs and that resilience had not yet been sufficiently trained. However, it is still noteworthy that HCPs were less resilient than controls immediately after the first COVID-19 wave in Italy; thus, our results may better reflect the detrimental consequences of the pandemic on the pre-existing resilient background of the HCPs.

Secondly, although we included a large sample of HCPs homogeneously distributed between the northern area and central or southern regions, we feel that the levels of resilience experienced by Italian HCPs during COVID-19 should be precautionarily compared to other populations. The main reason is that Italy was the first and hardest hit Western country, and it is therefore conceivable that the completely unexpected threat caused a greater burden compared to countries where national healthcare systems had more time to get prepared. Finally, the pool of predictors investigated in our survey is not sufficient to explain the entire phenomenon of the development of resilience in HCP; many other variables, such as coping strategies, emotional well-being, level of perceived stress, social support, and personality traits may explain a significant part of resilience in HCP.

## 5. Conclusions

In conclusion, our survey suggests that after the first COVID-19 wave, Italian HCPs had lower levels of resilience than the general population. Resilience levels in HCPs were negatively correlated with individual factors such as degree of anxiety and depression and environmental factors such as lack of adequate PPE. Furthermore, increasing BMI in males and working in COVID-19-free settings in females were significant predictors of having low resilience. In this context, promoting resilience among HCPs can represent an approach to counterbalance the higher-than-average levels of stress associated with extraordinary workload and reduce the risk of staff burnout. To this aim and in regards to the ongoing pandemic, several approaches have already been demonstrated to promote the development of resilience in HCPs, such as Mindfulness-Based Stress Reduction (MBSR) programs, interventions based on cognitive behavioral therapy (CBT) principles, and training and workshops aimed at cultivating self-reflection, self-regulation, relaxation, self-care, work-related stress reduction, and problem-focused learning [53]. These techniques can be delivered either through face-to-face groups or web-based platforms and therefore may be quickly implemented in workplaces to support HCPs during the ongoing second wave of the pandemic.

## Figures and Tables

**Table 1:** General characteristics of the study sample.

		COVID-19-Free Setting (*n* = 195) ^A^	Indirect COVID-19 Care (*n* = 451) ^B^	Direct COVID-19 Care (*n* = 363) ^C^	Controls (*n* = 375) ^D^	*p*-Value
Age, years		43.78 ± 13.12	43.37 ± 12.05	40.88 ± 11.23	48.39 ± 13.66 ^A,B,C^	<0.001
Gender, *n* (%)	Male	61 (31.3)	160 (35.5)	132 (36.4)	133 (35.5)	0.670
	Female	134 (68.7)	291 (64.5)	231 (63.6)	242 (64.5)	
Body mass index, kg/m^2^		24.12 ± 3.85	24.05 ± 3.85	23.82 ± 3.90	24.41 ± 3.79	0.218
Marital status, *n* (%)	Single	67 (34.4)	180 (39.9)	143 (39.4)	123 (32.8)	<0.001
	Married	109 (55.9)	241 (53.4)	188 (51.8)	212 (56.5)	
	Divorced	10 (5.1)	26 (5.8)	32 (8.8)	33 (8.8)	
	Widowed	9 (4.6)	4 (0.9)	0 (0)	7 (1.9)	
Smoking habits, *n* (%)	Never smoker	120 (61.5)	270 (59.9)	222 (61.2)	184 (49.1) ^A,B,C^	0.001
	Former smoker	50 (25.6)	96 (21.3)	65 (17.9)	104 (27.7) ^C^	
	Current smoker	25 (12.8)	85 (18.8)	76 (20.9)	87 (23.2) ^A^	
Geographic area, *n* (%)	Northern Italy	55 (28.2)	256 (56.8)	250 (68.9)	228 (60.8) ^A^	<0.001
	Southern and Central Italy	140 (71.8)	195 (43.2)	113 (31.1)	147 (39.2) ^A^	
Chronic comorbidities, *n* (%)		76 (39.0)	151 (33.5)	93 (25.6)	134 (35.7) ^C^	0.004
HCP role, *n* (%)	Physician	100 (51.3) ^#,B,C^	323 (71.6)	254 (70.0)	-	<0.001
	Nurse	34 (17.4) ^#^	71 (15.7)	76 (20.9)	-	
	Other HCP	61 (31.3) ^#,B,C^	57 (12.6)	33 (9.1)	-	
Medical specialty area, *n* (%)	Clinical	24 (12.3) ^#,B,C^	105 (23.3)	105 (28.9)	-	<0.001
	Surgical	9 (4.6) ^#,B,C^	78 (17.3)	44 (12.1)	-	
	Intensive Care	0 (0) ^#,B,C^	22 (4.9)	75 (20.7)	-	
	Radiology	1 (0.5) ^#,B^	46 (10.2)	10 (2.8)	-	
	Other medical specialties	46 (23.6) ^#,B,C^	48 (10.6)	8 (2.2)	-	
	General practice	28 (14.4^) #,C^	37 (8.2)	17 (4.7)	-	
COVID-19 screening, *n* (%)	Never	99 (50.8) ^#,B,C^	86 (19.1)	18 (5.0)	375 (100.0) ^A,B,C^	<0.001
	Occasionally	82 (42.1) ^#,B,C^	240 (53.2)	221 (60.9)	0 (0) ^A,B,C^	
	Regularly	14 (7.2) ^#,B,C^	125 (27.7)	124 (34.2)	0 (0) ^A,B,C^	
Symptomatic COVID-19, *n* (%)	No	179 (91.8) ^#,C^	384 (85.1)	307 (84.6)	375 (100.0) ^A,B,C^	<0.001
	Uncertain	14 (7.2)	42 (9.3)	31 (8.5)	0 (0) ^A,B,C^	
	Yes	2 (1.0) ^#,B,C^	25 (5.5)	25 (6.9)	0 (0) ^B,C^	
COVID-19 duration, days		1.13 ± 5.70	2.81 ± 9.53	2.11 ± 7.00	-	0.049
PPE satisfaction, 5-point Likert-type scale		2.58 ± 1.39 ^#,B,C^	2.81 ± 1.18	3.41 ± 1.16	-	<0.001

Data are presented as mean ± standard deviation or number (percentage) as appropriate. *p*-values for ANOVA analyses are reported. The superscript letter indicates the differences between individual groups in Bonferroni post-hoc analyses. In detail, the superscript letter indicates the presence of a statistically significant difference between the group in the column where the letter is reported and the group represented by the superscript letter itself. Each group is represented by a superscript letter shown in the first row. In particular: group A: COVID-19-Free Setting; group B: Indirect COVID-19 Care; group C: Direct COVID-19 Care; group D: Controls. ^#^ Analysis performed on subgroup of HCPs only. Legend: COVID-19, coronavirus disease 2019; HCP, healthcare professional; PPE, personal protective equipment.

## Supplementary Materials

The following are available online at https://www.mdpi.com/2076-328X/10/12/183/s1, Table S1: structure of the survey; Table S2: Scoring of individual 14-items Resilience Scale (RS14) items in healthcare professionals (HCP) and control individuals.
