# Supplementary material for: Levels and Factors Associated with Resilience in Italian Healthcare Professionals during the COVID-19 Pandemic: A Web-Based Survey
Source: Behav Sci (Basel). 2020 Nov 29;10(12):183. doi: 10.3390/bs10120183 (PMC7760580; doi:10.3390/bs10120183)
Supplement: Supplementary file 1 [file behavsci-10-00183-s001.pdf]

Supplementary information

Table S1. Structure of the survey. Legend: COVID-19, coronavirus disease 2019; HCP, healthcare professionals.

Original question (Italian)	English translation	Response allowed	Target population
Section 1: Professional, social and demographic aspects			
Nell'ospedale dove lavora sono stati ricoverati pazienti COVID-19 o è medico di medicina generale/continuità assistenziale ed ha lavorato a contatto con pazienti COVID-19?	Were COVID-19 patients admitted in the hospital where you work? Or are you a general practitioner working with COVID-19 patients?	Yes/ No	Only HCP
Ha lavorato o sta lavorando in un reparto dedicato al COVID-19?	Did you work, or are you working, in a COVID-19 unit?	Yes/ No	Only HCP
Che professione svolge in ambito sanitario?	What is your role in the health care system?	Physician/ Nurse/ Another role	Only HCP
Se medico, in che area disciplinare lavora?	If you are a physician, what is your specialty?	I'm not a physician/ General Medicine/ Clinical specialty/ Surgical specialty/Intensive care/ Radiology/ Other (open answer)	Only HCP
E' stato mai sottoposto a esami finalizzati a valutare la sua esposizione al virus?	Have you ever been screened for COVID-19?	Yes, regularly/ Yes, occasionally/ No, never	HCP and controls
E' risultato mai positivo ad uno di questi esami? (può selezionare più di una risposta)	Have you ever tested positive to one or more of these tests? (more answers allowed)	Yes, IgG serology/ Yes, IgM serology/ Yes, nasopharyngeal swab/ No	HCP and controls
Ha sviluppato malattia sintomatica?	Did you have symptomatic disease?	Yes/ No/ I had uncertain symptoms	HCP and controls
Quali di questi sintomi ha sviluppato? (può selezionare più risposte)	Which of these symptoms did you have? (more answers allowed)	Cough/ Fever/ Dyspnoea/ Sore throat/ Fatigue/ Muscle pain/ Joint pain/ Loss of smell/ Loss of taste/ Runny nose/ Other (open answer)	HCP and controls
Quanti giorni è durata la sua malattia?	How many days did your symptoms last?	Open answer	HCP and controls
Riguardo al suo setting assistenziale, ritiene di essere soddisfatto dei dispositivi di protezione individuale forniti dalla sua azienda?	About your work setting, do you think to be satisfied with the personal protective equipment provided by your employer?	0 (min) to 5 (max) Likert scale	Only HCP
In che regione abita?	In which region do you live?	Abruzzo/ Basilicata/ Calabria/ Campania/ Emila- Romagna/ Friuli-Venezia-Giulia/ Lazio/ Liguria/ Lombardia/ Marche/ Molise/ Piemonte/ Puglia/ Sardegna/ Sicilia/ Toscana/ Trentino-Alto Adige/ Umbria/ Valle d'Aosta/ Veneto	HCP and controls
Di che sesso è?	What is your sex?	Male/ Female	HCP and controls

Quanti anni ha?	What is your age?	Open answer	HCP and controls
Indichi gentilmente il suo peso	What is your weight?	Open answer	HCP and controls
Indichi gentilmente la sua altezza	What is your height?	Open answer	HCP and controls
E' fumatore?	Do you smoke?	No, I have never smoked<br>No, but I'm a former smoker<br>Yes, I'm a smoker	HCP and controls
Qual è il suo stato civile?	What is your marital status?	Married/ Single/ Divorced/ Widowed	HCP and controls
Soffre di patologie croniche? Può gentilmente indicare quali?	Do you have any chronic disease? Can you please write which?	Open answer	HCP and controls
Assume cronicamente dei farmaci? Può gentilmente indicare quali?	Are you under chronic therapies? Can you please write which?	Open answer	HCP and controls
Section 2: 14-item Resilience Scale (RS14)			
Item 1			HCP and controls
Item 2			HCP and controls
Item 3			HCP and controls
Item 4			HCP and controls
Item 5			HCP and controls
Item 6			HCP and controls
Item 7			HCP and controls
Item 8			HCP and controls
Item 9			HCP and controls
Item 10			HCP and controls
Item 11			HCP and controls
Item 12			HCP and controls
Item 13			HCP and controls
Item 14			HCP and controls
Section 3: Beck's Depression Inventory-II (BDI-II)			
Item 1			HCP and controls
Item 2			HCP and controls
Item 3			HCP and controls
Item 4			HCP and controls

Item 5	HCP and controls
Item 6	HCP and controls
Item 7	HCP and controls
Item 8	HCP and controls
Item 9	HCP and controls
Item 10	HCP and controls
Item 11	HCP and controls
Item 12	HCP and controls
Item 13	HCP and controls
Item 14	HCP and controls
Item 15	HCP and controls
Item 16	HCP and controls
Item 17	HCP and controls
Item 18	HCP and controls
Item 19	HCP and controls
Item 20	HCP and controls
Item 21	HCP and controls
Section 4: Hospital Anxiety and Depression Scale (HADS)	
Item 1	HCP and controls
Item 2	HCP and controls
Item 3	HCP and controls
Item 4	HCP and controls
Item 5	HCP and controls
Item 6	HCP and controls
Item 7	HCP and controls
Item 8	HCP and controls
Item 9	HCP and controls
Item 10	HCP and controls

Item 11	HCP and controls
Item 12	HCP and controls
Item 13	HCP and controls
Item 14	HCP and controls

Legend: COVID-19, coronavirus disease 2019; HCP, healthcare professionals.

Table S2. Scoring of individual 14-items Resilience Scale (RS14) items in healthcare professionals (HCP) and control individuals. Values with different subscript letter in each row have $p < 0.05$ for equality test between couple of columns (adjusted with Bonferroni correction).

RS14		Controls		HCP		
**Item**	**Score**	**Count**	**%**	**Count**	**%**	***p*-value**
	1	1_a	0.3%	9_a	0.9%	
	2	1_a	0.3%	11_a	1.1%	
	3	8_a	2.1%	37_a	3.7%	
1	4	32_a	8.5%	136_b	13.5%	0.004
	5	77_a	20.5%	230_a	22.8%	
	6	128_a	34.1%	326_a	32.3%	
	7	128_a	34.1%	260_b	25.8%	
	1	4_a	1.1%	0[1]	0.0%	
	2	4_a	1.1%	15_a	1.5%	
	3	11_a	2.9%	31_a	3.1%	
2	4	31_a	8.3%	121_b	12.0%	0.015
	5	87_a	23.2%	235_a	23.3%	
	6	132_a	35.2%	356_a	35.3%	
	7	106_a	28.3%	251_a	24.9%	
	1	12_a	3.2%	51_a	5.1%	
	2	21_a	5.6%	82_a	8.1%	
	3	44_a	11.7%	130_a	12.9%	
3	4	69_a	18.4%	242_b	24.0%	<0.001
	5	92_a	24.5%	278_a	27.6%	
	6	92_a	24.5%	154_b	15.3%	
	7	45_a	12.0%	72_b	7.1%	
	1	4_a	1.1%	14_a	1.4%	
	2	9_a	2.4%	38_a	3.8%	
	3	24_a	6.4%	81_a	8.0%	
4	4	38_a	10.1%	160_b	15.9%	0.008
	5	68_a	18.1%	203_a	20.1%	
	6	111_a	29.6%	270_a	26.8%	
	7	121_a	32.3%	243_b	24.1%	
	1	9_a	2.4%	9_b	0.9%	
	2	17_a	4.5%	38_a	3.8%	
	3	20_a	5.3%	82_a	8.1%	
5	4	50_a	13.3%	139_a	13.8%	0.016
	5	83_a	22.1%	287_b	28.4%	
	6	116_a	30.9%	279_a	27.7%	
	7	80_a	21.3%	175_a	17.3%	
	1	3_a	0.8%	2_a	0.2%	
	2	4_a	1.1%	17_a	1.7%	
6	3	20_a	5.3%	31_b	3.1%	0.198
	4	30_a	8.0%	99_a	9.8%	
	5	76_a	20.3%	192_a	19.0%	

	6	112$_a$	29.9%	316$_a$	31.3%	
	7	130$_a$	34.7%	352$_a$	34.9%	
7	1	3$_a$	0.8%	8$_a$	0.8%	0.030
	2	7$_a$	1.9%	21$_a$	2.1%	
	3	10$_a$	2.7%	55$_b$	5.5%	
	4	42$_a$	11.2%	132$_a$	13.1%	
	5	72$_a$	19.2%	248$_b$	24.6%	
	6	122$_a$	32.5%	284$_a$	28.1%	
	7	119$_a$	31.7%	261$_b$	25.9%	
8	1	3$_a$	0.8%	7$_a$	0.7%	0.546
	2	7$_a$	1.9%	15$_a$	1.5%	
	3	10$_a$	2.7%	47$_a$	4.7%	
	4	41$_a$	10.9%	122$_a$	12.1%	
	5	82$_a$	21.9%	190$_a$	18.8%	
	6	114$_a$	30.4%	325$_a$	32.2%	
	7	118$_a$	31.5%	303$_a$	30.0%	
9	1	2$_a$	0.5%	4$_a$	0.4%	0.241
	2	5$_a$	1.3%	9$_a$	0.9%	
	3	13$_a$	3.5%	45$_a$	4.5%	
	4	36$_a$	9.6%	114$_a$	11.3%	
	5	90$_a$	24.0%	279$_a$	27.7%	
	6	115$_a$	30.7%	317$_a$	31.4%	
	7	114$_a$	30.4%	241$_b$	23.9%	
10	1	1$_a$	0.3%	7$_a$	0.7%	0.156
	2	4$_a$	1.1%	18$_a$	1.8%	
	3	11$_a$	2.9%	44$_a$	4.4%	
	4	29$_a$	7.7%	96$_a$	9.5%	
	5	67$_a$	17.9%	205$_a$	20.3%	
	6	124$_a$	33.1%	337$_a$	33.4%	
	7	139$_a$	37.1%	302$_b$	29.9%	
11	1	7$_a$	1.9%	15$_a$	1.5%	0.257
	2	5$_a$	1.3%	31$_a$	3.1%	
	3	21$_a$	5.6%	49$_a$	4.9%	
	4	36$_a$	9.6%	122$_a$	12.1%	
	5	83$_a$	22.1%	233$_a$	23.1%	
	6	109$_a$	29.1%	301$_a$	29.8%	
	7	114$_a$	30.4%	258$_a$	25.6%	
12	1	7$_a$	1.9%	21$_a$	2.1%	< 0.001
	2	7$_a$	1.9%	30$_a$	3.0%	
	3	12$_a$	3.2%	54$_a$	5.4%	
	4	29$_a$	7.7%	113$_a$	11.2%	
	5	55$_a$	14.7%	170$_a$	16.8%	
	6	86$_a$	22.9%	281$_a$	27.8%	
	7	179$_a$	47.7%	340$_b$	33.7%	
13	1	8$_a$	2.1%	10$_a$	1.0%	0.127
	2	1$_a$	0.3%	15$_a$	1.5%	
	3	10$_a$	2.7%	27$_a$	2.7%	
	4	40$_a$	10.7%	106$_a$	10.5%	
	5	62$_a$	16.5%	201$_a$	19.9%	
	6	122$_a$	32.5%	342$_a$	33.9%	
	7	132$_a$	35.2%	308$_a$	30.5%	
14	1	3$_a$	0.8%	7$_a$	0.7%	0.657
	2	7$_a$	1.9%	19$_a$	1.9%	
	3	9$_a$	2.4%	33$_a$	3.3%	
	4	31$_a$	8.3%	96$_a$	9.5%	
	5	94$_a$	25.1%	286$_a$	28.3%	

6	138a	36.8%	355a	35.2%
7	93a	24.8%	213a	21.1%
